# Pramipexole modulates fronto-subthalamic pathway in sequential working memory

**DOI:** 10.1038/s41386-022-01494-z

**Published:** 2022-11-09

**Authors:** Marcus Heldmann, Eliana Mönch, Antonia Kesseböhmer, Norbert Brüggemann, Thomas F. Münte, Zheng Ye

**Affiliations:** 1 Department of Neurology, University of Lübeck, Lübeck, 23538 Germany; 2 Institute of Psychology II, University of Lübeck, Lübeck, 23538 Germany; 3 Institute of Neurogenetics, University of Lübeck, Lübeck, 23538 Germany; 4 Institute of Neuroscience, Center for Excellence in Brain Science and Intelligence Technology, Chinese Academy of Sciences, Shanghai, 200031 China

**Keywords:** Cognitive control, Human behaviour

## Abstract

Brain dopamine may regulate the ability to maintain and manipulate sequential information online. However, the precise role of dopamine remains unclear. This pharmacological fMRI study examined whether and how the dopamine D2/3 receptor agonist pramipexole modulates fronto-subthalamic or fronto-striatal pathways during sequential working memory. This study used a double-blind, randomized crossover design. Twenty-two healthy male volunteers completed a digit ordering task during fMRI scanning after receiving a single oral dose of 0.5-mg pramipexole or placebo. The pramipexole effects on task performance, regional activity, activity pattern similarity, and functional connectivity were analyzed. Pramipexole impaired task performance, leading to less accurate and slower responses in the digit ordering task. Also, it downregulated the maintenance-related subthalamic and dorsolateral prefrontal activity, increasing reaction times for maintaining sequences. In contrast, pramipexole upregulated the manipulation-related subthalamic and dorsolateral prefrontal activity, increasing reaction time costs for manipulating sequences. In addition, it altered the dorsolateral prefrontal activity pattern similarity and fronto-subthalamic functional connectivity. Finally, pramipexole reduced maintenance-related striatal activity, which did not affect the behavior. This study confirms the role of the fronto-subthalamic pathway in sequential working memory. Furthermore, it shows that D2 transmission can regulate sequential working memory by modulating the fronto-subthalamic pathway.

## Introduction

Brain dopamine is known to regulate visuospatial working memory [1–3]. It may also regulate sequential working memory as patients with Parkinson’s disease (PD) exhibit difficulties in arranging items in a specific order [4, 5], understanding stories that are told backward [6, 7], and planning sequential steps to achieve goals [8–10]. To better understand the role of dopaminergic innervation, this study investigates whether and how the D2/3 receptor agonist pramipexole modulates the fronto-subthalamic hyperdirect or fronto-striatal indirect pathway during sequential working memory.

In visuospatial working memory, the dual-state theory of prefrontal dopamine function [11] assumes that a cortical D2-dominated state (e.g., D2 receptor activation) facilitates flexible manipulation, whereas a cortical D1-dominated state (e.g., D2 receptor blockade) favors persistent maintenance of information. The tonic-phasic dopamine hypothesis emphasizes a balance of tonic and phasic dopamine function in the frontal and subcortical regions [12, 13]. Namely, increased phasic and reduced tonic dopamine transmission subcortically and reduced dopamine concentration cortically (i.e., increased D2 and reduced D1 transmission) may facilitate flexible manipulation but impair persistent maintenance of information. In contrast, reduced phasic and increased tonic dopamine transmission subcortically and increased dopamine concentration cortically (i.e., increased D1 and reduced D2 transmission) may have opposite effects on visuospatial working memory [14].

Distinct roles of D1 *versus* D2 transmission in sequential working memory are less understood. Human pharmacological and genetic studies had inconsistent findings. In healthy adults, the D2 receptor antagonist sulpiride (D2 receptor blockade) can improve performance in reordering sequential items [15] but impair performance in planning sequential steps [16, 17]. In patients with PD, both the D2 receptor agonist bromocriptine (predominant D2 receptor activation) and levodopa (predominant D1 receptor activation) can improve performance in maintaining or reordering sequential items [18, 19]. The Val allele of the COMT Val^158^Met polymorphism (high COMT activity, increased D2 and reduced D1 transmission) improves performance in planning sequential steps [20]. But the Val allele of the BDNF Val^66^Met polymorphism (high BDNF secretion and increased D3 transmission) impairs performance in planning sequential steps [21]. These findings don’t fit well with current dopaminergic models of visuospatial working memory.

We have put forward a fronto-basal ganglia model for sequential working memory [22]. We assume that the prefrontal cortex encodes and retrieves sequential items through a competitive queuing mechanism [23–25]. The competitive queuing mechanism comprises a parallel planning layer, which represents the serial position of items as the relative strength of node activations, and a competitive choice layer, which forms reciprocal connections which the parallel planning layer and selects the node with the strongest activation. The basal ganglia may interact with the competitive choice layer to update the node activation in the parallel planning layer (e.g., inhibiting items that should be moved downward in the new order). This process may be promoted by inhibitory functions of the fronto-subthalamic hyperdirect and fronto-striatal indirect pathways.

**Fig. 1:**
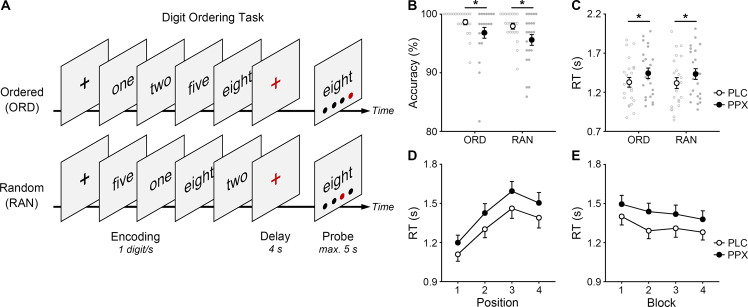
Digit ordering task and pramipexole effects on task performance. **A** Digit ordering task (translated from German). ORD ordered; RAN random. Individual data, group means and SEMs of (**B**) the accuracy and (**C**) reaction time (RT) for ORD and RAN trials under pramipexole (PPX) or placebo (PLC). Mean RTs and SEMs for different (**D**) serial positions or (**E**) experimental blocks.

We hypothesize that activation of D2/3 receptors regulates sequential working memory by modulating the fronto-subthalamic hyperdirect or fronto-striatal indirect pathway. D2 receptors are abundant in the striatum and also found in the subthalamic nucleus (STN) and frontal cortex [26, 27]. To test the hypothesis, we investigated the effects of a single oral dose of the D2/3 receptor agonist pramipexole on the fronto-subthalamic and fronto-striatal pathways using a double-blind, randomized crossover design. Each participant conducted a digit ordering task (Fig. 1A) [5, 28] during fMRI scanning twice, once under pramipexole and once under placebo. In the digit ordering task, participants memorized a sequence of ordered digits (ordered trials) or rearranged random digits in ascending order (random trials). The task distinguished between sequence maintenance (i.e., short-term memory processes identically involved in ordered and random trials) and manipulation (i.e., a cognitive process uniquely involved in random trials). The manipulation process was then isolated by subtracting ordered trials from random trials at brain and behavior levels (cognitive subtraction) [29]. First, we expected to replicate the manipulation-related regional activation over the dorsolateral prefrontal cortex (dlPFC), STN, and striatum. Second, we sought to detect the pramipexole effects on regional activity, prefrontal activity pattern similarity, and fronto-subthalamic functional connectivity. Third, we aimed to explore whether pramipexole-induced behavioral changes correlated with STN activity changes.

## Materials and methods

The study was approved by the ethics committee of the University of Lübeck following the Declaration of Helsinki. All participants signed written informed consent before participating in this study.

### Participants

We only recruited male volunteers to avoid problems that could arise with unknown pregnancies in female volunteers. Twenty-six healthy men participated (mean age 26.0 ± 4.1 years, range 20–38 years). They were right-handed and had normal or corrected-to-normal vision. None of them had a history of neurological or psychiatric disease. All of them were free of medication. Four participants were excluded because of excessive head motion (mean total displacement >1.5 mm, *n* = 1) or poor task performance (task accuracy <50%, *n* = 3).

### Study design

This study had a double-blind, randomized crossover design. Participants received pramipexole and placebo in separate sessions at least seven days apart (mean interval 40.4 ± 34.2 days). At each session, they received 10-mg domperidone in a non-blind fashion to antagonize nausea and other potential side effects induced by pramipexole [30]. One hour after the intake of domperidone, they received 0.5-mg pramipexole or placebo in a double-blind manner according to a randomization table. The dose of pramipexole was similar to that used in our previous work [31, 32] but less than that commonly used in patients with Parkinson’s disease. Twelve participants received pramipexole in the first session and the rest in the second session. Participants started fMRI scanning 2 h after the oral administration of pramipexole as the plasma concentration peaks about 2 h after intake [33]. No participant reported nausea or sleepiness in the pramipexole session.

### Digit ordering task

All participants conducted the digit ordering task during fMRI scanning (Fig. 1A). The task included interleaved 60 ordered trials and 64 random trials. Participants saw four different digits. They were asked to memorize the digits in ascending order over a short delay. In ordered trials, the digits were presented already in ascending order (e.g., 1-2-5-8). In random trials, the digits were fully randomized, and participants had to reorder them (e.g., 5-1-8-2). After the delay, participants saw a pair of digit and position. They were asked to judge whether the digit matched the position in the target order by pressing the yes/no buttons with their right hand.

### Analysis of task performance

We controlled behavioral data quality by monitoring premature (reaction time, RT, shorter than 0.1 s) and inattentive responses (RT longer than 3 SDs above the individual mean). Participants made no premature response and very few inattentive responses (pramipexole: 1.1%, placebo: 1.5%). The inattentive responses were excluded from further analysis.

First, we detected the pramipexole effects on accuracy and RT using ANOVAs with two factors (*p* < 0.05), Drug (pramipexole, placebo) and Trial Type (ordered, random). Second, we examined whether the pramipexole effect on RT persisted across serial positions using an ANOVA with three factors (*p* < 0.05), Drug (pramipexole, placebo), Target Position (four positions), and Trial Type (ordered, random). Third, we examined whether the pramipexole effect on RT persisted across experimental blocks using an ANOVA with three factors (*p* < 0.05), Drug (pramipexole, placebo), Block (four blocks), and Trial Type (ordered, random).

### Acquisition of MRI and fMRI data

Brain imaging data were acquired on a Siemens Magnetom Skyra 3 T MRI scanner with a 64-channel head coil. Structural T1-weighted images used a magnetization-prepared rapid gradient-echo sequence (208 sequential sagittal slices, repetition time 2300 ms, echo time 2.43 ms, inversion time 1100 ms, flip angle 8°, field of view 240 × 240 mm^2^, and spatial resolution 0.85 × 0.75 × 0.75 mm^3^).

Functional T2*-weighted images used a simultaneous multi-slice echo-planar imaging sequence with acceleration factor 4 (56 interleaved axial slices, repetition time 1000 ms, echo time 30 ms, flip angle 60°, field of view 204 × 204 mm^2^, and spatial resolution 3 × 3 × 3 mm^3^).

Field map images used a gradient echo sequence (46 axial slices, repetition time 489 ms, short echo time 4.92 ms, long echo time 7.38 ms, flip angle 60°, field of view 204 × 204 mm^2^, and spatial resolution 2.5 × 2.5 × 3.3 mm^3^).

### Preprocessing and analysis of fMRI data

fMRI data were processed using SPM12 (v7771, www.fil.ion.ucl.ac.uk/spm). A voxel displacement map was derived from the presubtracted phase and magnitude field map data for correcting geometric distortion of fMRI data. The first six images of each fMRI block were discarded to allow magnetization equilibration. All other fMRI images were corrected for slice acquisition time difference, realigned to the first image, corrected for geometric distortion, registered to the structural T1-weighted image, normalized to the Montreal Neurological Institute (MNI) coordinate system, smoothed with a Gaussian kernel of 6-mm full-width half-maximum, and filtered with a 128-s high-pass filter.

We controlled fMRI data quality by monitoring the scan-to-scan total displacement (mean total displacement <1.5 mm) [34], field map correction, and spatial normalization (visual inspection). The total displacement under pramipexole (0.62 mm) was similar to that under placebo (0.59 mm, paired *t* test, *t* < 1).

First, we replicated the manipulation-related regional activation. The general linear model convolved a design matrix with a canonical hemodynamic response function at the subject level. The design matrix included correct and incorrect ordered and random trials as separate regressors. Each trial was time-locked to its onset. The total displacement was included as a nuisance regressor. Classical parameter estimation was applied with a one-lag autoregressive model. The manipulation-related activation was defined as correct random *versus* ordered trials. A whole-brain one-sample *t* test was conducted at the group level for each drug (voxel-level *p* < 0.001, cluster-level *p* < 0.05 familywise error correction).

Second, we detected pramipexole effects on regional activity in fronto-subthalamic and fronto-striatal pathways. The left dlPFC and left striatal regions were derived from a meta-analysis of 1091 fMRI studies on working memory (NeuroSynth) [35]. However, the working memory map did not cover the STN. Therefore, the left STN region was derived from a human basal ganglia atlas [36]. Finite impulse response (FIR) timecourses of ordered and random trials (i.e., model-based estimates of fMRI signals) were extracted from each region. To examine pramipexole effects on the maintenance-related regional activity, we computed mean amplitudes of the FIR timecourses between 5–12 s post-onset for ordered trials (i.e., delay and probe stages), and entered them into a one-tailed paired *t* test for each region (pramipexole<placebo, *p* < 0.02 Bonferroni correction for three tests). To examine pramipexole effects on the manipulation-related regional activity, we computed difference waves between the FIR timecourses of random trials and those of ordered trials, calculated mean amplitudes of the manipulation-related difference waves between 5–12 s post-onset, and entered them into a one-tailed paired *t* test for each region (pramipexole>placebo, *p* < 0.02 Bonferroni correction for three tests).

Third, we explored relationships between behavior and fronto-subthalamic and fronto-striatal pathways. We examined whether the pramipexole-induced RT change (ordered trials) correlated with the maintenance-related regional activity change in the dlPFC, STN, or striatum (stepwise regression in the IBM SPSS, *p* < 0.05). The pramipexole-induced maintenance-related regional activity change was estimated from difference waves between the FIR timecourses of ordered trials under pramipexole and those under placebo, and measured as the maximal negative activity change (Fig. 3A, blue line). We examined whether the pramipexole-induced manipulation-related RT cost change (random *versus* ordered trials) correlated with the manipulation-related regional activity change in the dlPFC or STN (stepwise regression, *p* < 0.05). The pramipexole-induced manipulation-related regional activity change was estimated from difference waves between the manipulation-related difference waves under pramipexole and those under placebo, and measured as the maximal positive activity change (Fig. 3A, red line).

Fourth, we detected pramipexole effects on prefrontal activity pattern similarity. Beta values of ordered and random trials (i.e., model-based estimates of fMRI signals without time dimension) were extracted from dlPFC voxels and sorted spatially according to MNI coordinates. Pearson correlation coefficients were computed between the betas of ordered trials and those of random trials, normalized using Fisher’s transformation, and entered into a paired *t* test (*p* < 0.05).

Finally, we detected pramipexole effects on fronto-subthalamic functional connectivity. Raw fMRI signals were extracted from the left dlPFC and left STN regions and demeaned. Pearson correlation coefficients were computed between the dlPFC and STN signals, normalized using Fisher’s transformation, and entered into a paired *t* test (*p* < 0.05).

## Results

### Pramipexole impaired task performance

Pramipexole impaired digit ordering task performance (Fig. 1B, C). For accuracy, a main effect of Drug (ANOVA, *F*(1,21) = 8.95, *p* = 0.007, *η*^2^ = 0.30) and a marginal main effect of Trial Type were found (*F*(1,21) = 3.75, *p* = 0.066, *η*^2^ = 0.15), but no interaction (*F* < 1). For reaction time, a main effect of Drug was found (ANOVA, *F*(1,21)=6.70, *p* = 0.017, *η*^2^ = 0.24), but no main effect of Trial Type or interaction (*F*s < 1). Participants responded less accurately and more slowly under pramipexole than under placebo. We did not obtain a group-level pramipexole effect on manipulation-related RT cost (random *versus* ordered trials) due to individual differences in drug response. Therefore, we applied individual difference analysis later (Fig. 3C, D).

The pramipexole effect on RT persisted across serial positions and experimental blocks (Fig. 1D, E). For serial positions, main effects of Drug (ANOVA, *F*(1,21) = 6.77, *p* = 0.017, *η*^2^ = 0.24) and Target Position were found (*F*(3,63) = 38.18, *p* < 0.001, *η*^2^ = 0.65), but no interaction (*F* < 1). For experimental blocks, main effects of Drug (ANOVA, *F*(1,21) = 6.51, *p* = 0.017, *η*^2^ = 0.24) and Block were found (*F*(3,63) = 8.34, *p* = 0.001, *η*^2^ = 0.28), but no interaction (*F* < 1). Participants showed serial position effects (primacy and recency effects) and a learning effect. The pramipexole effect was evenly distributed across different serial positions and experimental blocks.

The pramipexole effect on RT was unlikely due to sleepiness or other side effects of the drug. Participants responded equally fast in an independent decision-making task under pramipexole *versus* placebo (see Supplementary information).

### Pramipexole altered regional activity in fronto-subthalamic and fronto-striatal pathways

**Fig. 2:**
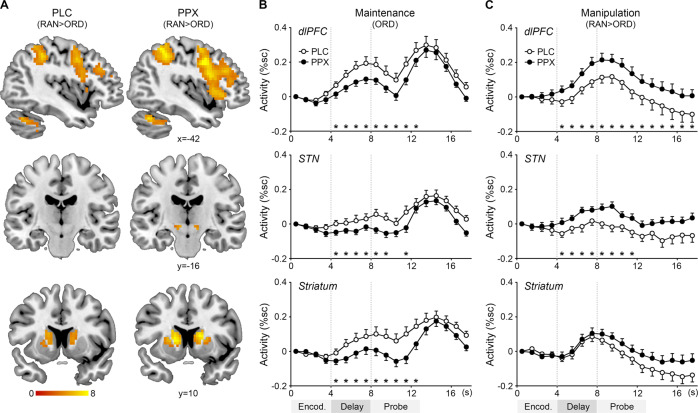
Pramipexole effects on regional activity. **A** Manipulation-related regional activation (random *versus* ordered trials, RAN > ORD) under pramipexole (PPX) or placebo (PLC). The color bar indicates *t* values. Coordinates are in the MNI space. Mean FIR timecourses and SEMs of (**B**) the maintenance-related regional activity and (**C**) the manipulation-related regional activity. dlPFC dorsolateral prefrontal cortex, STN subthalamic nucleus, %sc percent signal change, asterisks, *p* < 0.05.

Table 1 and Fig. 2A show manipulation-related regional activation under each drug. The manipulation-related regional activation in the dlPFC, STN, and striatum was numerically larger under pramipexole than under placebo. The visual inspection was confirmed by region of interest analysis.

**Table 1:** Ordering-related regional activation (random *versus* ordered trials).

Region	Side	Placebo		Pramipexole	
		Peak (*t* value)	Cluster size (voxels)	Peak (*t* value)	Cluster size (voxels)
Dorsolateral prefrontal cortex	L	6.90	308	10.24	516
	R	7.22	310	7.74	395
Ventrolateral prefrontal cortex	L	5.99	167	9.80	521
Dorsomedial prefrontal cortex	L/R	6.66	331	10.96	540
Parietal lobule	L	10.75	466	9.83	566
	R	7.60	294	7.96	387
Striatum	L	6.18	81	8.43	146
	R	5.42	61	8.42	141
Globus pallidus	L	4.94	28	6.77	38
	R	5.47	26	5.14	28
Subthalamic nucleus	L	–	–	4.84	6
	R	–	–	4.77	5
Thalamus	L	7.10	173	6.33	214
	R	6.83	134	7.41	164
Cerebellum	L	7.75	266	8.51	279
	R	10.18	482	11.24	638

*MNI* Montreal Neurological Institute coordinate system, *L* *left*, *R* right.

Figure 2B shows pramipexole effects on the maintenance-related regional activity (ordered trials, [5 12] s post-onset). Pramipexole reduced the maintenance-related regional activity in the left dlPFC (*t*(21) = −3.05, *p* = 0.003), left STN (*t*(21) = −2.50, *p* = 0.010), and left striatum (*t*(21) = −2.86, *p* = 0.005).

Figure 2C shows pramipexole effects on the manipulation-related regional activity (random *versus* ordered trials, [5 12] s post-onset). Pramipexole increased the manipulation-related regional activity in the left dlPFC (*t*(21) = 3.13, *p* = 0.003) and left STN (*t*(21) = 2.62, *p* = 0.008), but not in the left striatum (*t* < 1).

We focused on the left side as previous work showed a left-lateralized neural system for sequential working memory [5, 22]. The right side showed similar tendencies (see Supplementary information).

### Relationships between task performance and fronto-subthalamic pathway

**Fig. 3:**
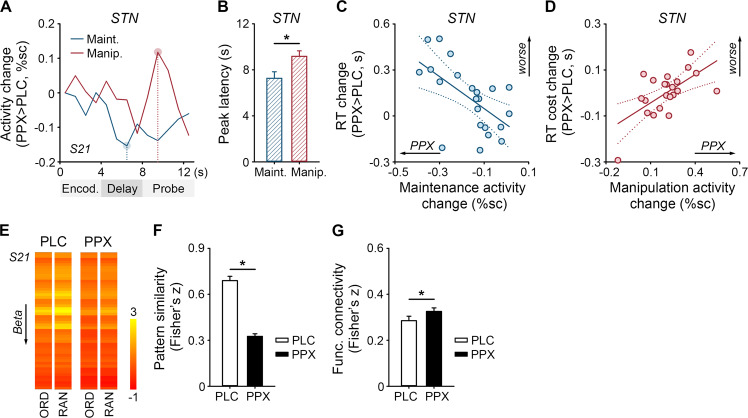
Relationships between behavior and fronto-subthalamic pathway. **A** The pramipexole-induced (PPX > PLC) maintenance-related regional activity change and pramipexole-induced manipulation-related regional activity change from the subthalamic nucleus (STN) of a representative subject (S21). Maximum activity changes (peaks) are marked. %sc, percent signal change. **B** Means and SEMs of the peak latency in the STN. Asterisks, *p* < 0.05. **C** Correlation between the pramipexole-induced reaction time (RT) change and maintenance-related STN activity change. **D** Correlation between the pramipexole-induced manipulation-related RT cost change and manipulation-related STN activity change. Solid lines, *p* < 0.05; dotted lines, 95% confidence intervals. **E** Prefrontal activity patterns of the representative subject (50/300 voxels). The color bar indicates beta values. Means and SEMs of (**F**) the prefrontal activity pattern similarity and (**G**) fronto-subthalamic functional connectivity.

Figure 3A shows the pramipexole-induced maintenance- and manipulation-related STN activity changes of a representative subject. The pramipexole-induced maintenance-related STN activity change peaked earlier than the manipulation-related STN activity change (paired *t* test, *t*(21) = −2.39, *p* = 0.026, Fig. 3B).

The pramipexole-induced RT change (ordered trials) negatively correlated with the maintenance-related STN activity change (Fig. 3C). The stepwise regression model (*F*(1,20)=8.66, *p* = 0.008, *R*^2^ = 0.30) included the pramipexole-induced maintenance-related regional activity change in the left STN (*t* = −2.94, *p* = 0.008) but not that in the dlPFC or striatum (*p*s > 0.24). Participants with greater maintenance-related STN (but not dlPFC or striatal) activity reduction showed larger RT increases under pramipexole *versus* placebo.

The pramipexole-induced RT cost change (random *versus* ordered trials) positively correlated with the manipulation-related STN activity change (Fig. 3D). The stepwise regression model (*F*(1,20) = 10.18, *p* = 0.005, *R*^2^ = 0.34) included the pramipexole-induced manipulation-related regional activity change in the left STN (*t* = 3.19, *p* = 0.005) but not that in the dlPFC (*p* = 0.647). Participants with greater manipulation-related STN (but not dlPFC) activity promotion showed larger RT cost increases under pramipexole *versus* placebo.

### Pramipexole reduced prefrontal activity pattern similarity

Figure 3E shows the prefrontal activity pattern of a representative subject under each drug. Pramipexole reduced the similarity between the dlPFC activity pattern of ordered trials and that of random trials (paired *t* test, *t*(21) = −13.91, *p* < 0.001, Fig. 3F). However, the pramipexole-induced prefrontal activity pattern similarity change did not correlate with the RT or RT cost changes (*p*s > 0.62).

### Pramipexole enhanced fronto-subthalamic functional connectivity

Pramipexole enhanced the functional connectivity between the left dlPFC and left STN (paired *t* test, *t*(21) = 2.20, *p* = 0.039, Fig. 3G). However, the pramipexole-induced fronto-subthalamic functional connectivity change did not correlate with the RT or RT cost changes (*p*s > 0.65).

## Discussion

Brain dopamine may regulate sequential working memory. Dopamine deficiency in PD leads to difficulties in diverse sequencing tasks that rely on the persistent maintenance and flexible manipulation of sequential information [4, 6, 8]. To better understand the role of dopaminergic innervation, we investigated whether activation of D2/3 receptors modulates the fronto-basal ganglia loops during sequential working memory using pharmacological fMRI with a digit ordering task.

A single oral dose of the D2/3 receptor agonist pramipexole led to less accurate and slower responses in the digit ordering task. Also, it downregulated the dlPFC, STN, and striatal regional activity for maintaining sequences (ordered trials). In contrast, pramipexole upregulated the dlPFC and STN regional activity for manipulating sequences (random *versus* ordered trials). Additionally, it altered the dlPFC activity pattern similarity and fronto-subthalamic functional connectivity. More importantly, the pramipexole-induced maintenance-related STN activity change predicted the RT change for maintaining sequences. The pramipexole-induced manipulation-related STN activity change predicted the RT cost change for manipulating sequences.

### Fronto-basal ganglia loops and sequential working memory

In the fronto-basal ganglia model for sequential working memory [22], we proposed that the basal ganglia may interact with the prefrontal cortex to realize sequence manipulation (e.g., inhibiting items that should be moved downward in the new order). In particular, we proposed that the process may be promoted by inhibitory functions of the fronto-subthalamic hyperdirect and fronto-striatal indirect pathways.

**Fig. 4:**
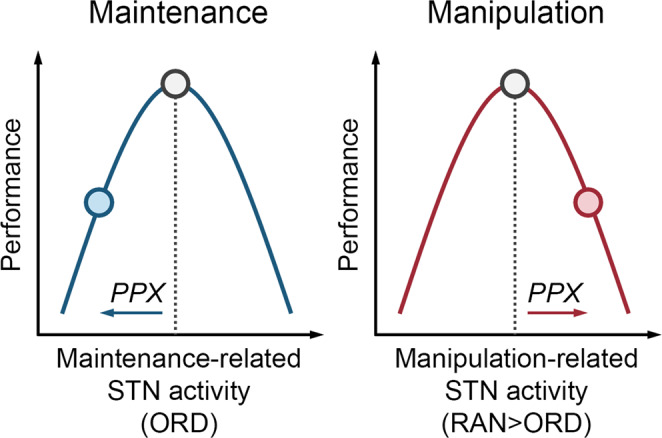
Non-linear relationships between STN and sequential working memory. The non-linear relationship is presented as a quadratic function for simplicity. Other non-linear functions are possible [39]. STN subthalamic nucleus, PPX pramipexole.

This study confirmed the role of the fronto-subthalamic pathway in sequential working memory. The relationship between D2-receptor modulated STN activity and sequential working memory is non-linear: both insufficient and excessive STN activity impairs sequential working memory [14, 37, 38]. Figure 4 is a schematic diagram of the relationship. Maintenance and manipulation are presented as two separate curves. Healthy adults have optimal STN activity in the maintenance and manipulation curves. D2 receptor activation (pramipexole) reduces the maintenance-related STN activity, leading to suboptimal performance in maintaining sequences. It also increases the manipulation-related STN activity, leading to suboptimal performance in manipulating sequences. It is worth noting that, non-linear relationships have been observed between brain dopamine and behavior across cognitive domains [39]. Further research with multidrug or multidose is needed to better describe the non-linear relationship.

### D2/3 transmission in the fronto-subthalamic pathway

A novel finding is that D2 receptor activation can downregulate the maintenance-related STN and dlPFC activity and dlPFC activity pattern similarity but upregulate the manipulation-related STN and dlPFC activity and fronto-subthalamic functional connectivity. Although the STN and frontal cortex have a much lower density of D2 receptors than the striatum [26, 27], D2 receptor activation can significantly alter the functions of STN and prefrontal cortex [40–43].

Mechanisms underlying the observed maintenance-related activity downregulation and manipulation-related activity upregulation remain unclear. For the prefrontal cortex, the maintenance-related activity change may result from decreased regional cerebral blood flow. Black et al. [43] showed that medium to high doses of pramipexole (50–500 μk/kg) could reduce regional cerebral blood flow over the lateral and medial prefrontal cortex in non-human primates. However, Black et al. did not examine the STN.

Futhermore, D2 transmission can modulate membrane resonance of STN neurons and high-voltage spindles (HVSs) in the fronto-basal ganglia loops. Yang et al. [44] showed that in rat brain slices, D2 receptor blockade inhibited theta-frequency membrane resonance of STN neurons by suppressing hyperpolarization-activated cyclic nucleotide-gated channels. In rats, STN D2 receptor blockade increased the HVS power in the frontal cortex and globus pallidus as well as the HVS coherence between the frontal cortex and globus pallidus. However, Yang et al. did not examine D2 receptor activation. Further research with animal models is needed to understand the exact mechanisms of D2 transmission in the fronto-basal ganglia loops.

### D2 transmission and sequential working memory

A second novel finding is that D2 receptor activation can negatively affect sequential working memory. Pramipexole can impair sequence maintenance by inhibiting maintenance-related STN activity and impair sequence manipulation by enhancing manipulation-related STN activity. Another possibility is that pramipexole slowed down information processing in general, although sleepiness and other side effects of pramipexole have been minimized. Additionally, despite no direct correlation, pramipexole-induced dlPFC activity pattern similarity or fronto-subthalamic functional connectivity change might produce behavioral deficits.

This finding is not entirely consistent with previous pharmacological studies and dopaminergic models of visuospatial working memory. For example, the D2 receptor agonist bromocriptine and the partial agonist aripiprazole can increase the dlPFC activity for maintaining visual objects or spatial locations [45, 46], even though the drugs do not always lead to behavioral improvement [30, 47, 48]. Moreover, D2 receptor activation is assumed to boost flexible manipulation of visuospatial information through a cortical D2-dominated state (the dual-state theory) [11] or increased phasic and reduced tonic striatal dopamine transmission (the tonic-phasic hypothesis) [13, 38].

The inconsistency between our observation and previous studies is not unexpected. The cognitive and neural mechanisms that encode and retrieve sequential information may differ from those that encode and retrieve visuospatial information. For example, Ranganath and colleagues found that frontal theta power was enhanced for maintaining serial positions over visual features of the same item in healthy adults [49, 50]. Other researchers found that the lateral prefrontal cortex and intraparietal sulcus were more activated for maintaining serial positions than visual features in healthy adults and children [51–53].

### D2 transmission in the fronto-striatal pathway

A third finding is that D2 receptor activation can downregulate the striatal activity for maintaining (but not manipulating) sequences. This finding is compatible with our previous results. First, the striatum is hypo-activated in PD patients medicated with D2/3 receptor agonists and levodopa [5] but not in untreated patients (unpublished data). Second, the manipulation-related striatal activity is unrelated to daily exposure to D2/3 receptor agonists in medicated patients with PD [5].

D2 receptors are abundant in the striatum [26, 27]. Pramipexole may downregulate maintenance-related striatal activity by reducing tonic striatal dopamine transmission. Maruya et al. [54] showed that in rats, pramipexole and other D2 receptor agonists can suppress excessive dopamine releases and hyperactivity induced by the L-type calcium channel agonist Bay X 8644. Reduced tonic dopamine transmission is often assumed to be accompanied by enhanced phasic dopamine transmission, potentially leading to greater manipulation-related striatal activity. However, this is not the case in our study.

The role of striatal D2 transmission might differ in sequential *versus* visuospatial working memory. In visuospatial working memory, striatal D2 transmission is assumed to support working memory updating and mental set shifting [38, 55, 56]. For example, Cools et al. [46] found that D2 receptor activation increased the switching-related striatal activity and reduced behavioral switching costs in high-impulsive adults. Li et al. [57] showed that the G allele of Taq1A polymorphism (increased striatal D2 receptor density) correlated with greater striatal activity and higher performance accuracy for updating working memory contents in older adults. Further research is needed to confirm the distinct roles of striatal D2 transmission in sequential *versus* visuospatial working memory.

### Limitations

A major limitation is that female volunteers were excluded, and women might respond differently to pramipexole during sequential working memory. As there are concerns about giving pramipexole to women of childbearing age [58, 59], the local ethics committee approved the experimental use of pramipexole only in men but not in women. A second major limitation is that the plasma concentration of pramipexole was not measured. This study could not infer a link between the bioavailability of pramipexole and changes in the brain and behavior. Third, STN is a small structure, but different subregions of the STN may contribute differently to sequential working memory [22]. The spatial resolution of 3 T MRI is limited. Further research with ultra-high field MRI is needed to confirm our findings and determine the contribution of each STN subregion [60]. In addition, STN is a node in the fronto-striatal indirect pathway. This study could not exclude the possibility that the pramipexole-induced maintenance-related STN activity change is due to modulatory effects on the striatum.

## Conclusions

This study shows that D2 receptor activation could modulate the fronto-subthalamic pathway during sequential working memory. The D2/3 receptor agonist pramipexole downregulated the maintenance-related STN and dlPFC activity, increasing reaction times for maintaining sequences. In contrast, pramipexole upregulated the manipulation-related STN and dlPFC activity, increasing reaction time costs for manipulating sequences. In addition, pramipexole altered the dlPFC activity pattern similarity and fronto-subthalamic functional connectivity. It also reduced maintenance-related striatal activity, which did not affect the behavior. This study confirmed the role of the fronto-subthalamic pathway in sequential working memory. Furthermore, it shows that both insufficient and excessive STN activity can impair sequential working memory. Our finding does not fit neatly with existing dopaminergic models of working memory and therefore suggests the need for more work in this area.

## Supplementary information

Supplement
