# Supplementary material for: Pramipexole modulates fronto-subthalamic pathway in sequential working memory
Source: Neuropsychopharmacology. 2022 Nov 9;48(5):716–23. doi: 10.1038/s41386-022-01494-z (PMC10066371; doi:10.1038/s41386-022-01494-z)
Supplement: Supplementary file 1 — Supplement [file 41386_2022_1494_MOESM1_ESM.docx]

Supplement for

**Pramipexole modulates fronto-subthalamic pathway in sequential working memory**

Marcus Heldmann,^1,2^ Eliana Mönch,^1^ Antonia Kesseböhmer,^1^ Norbert Brüggemann,^1,3^ Thomas F. Münte,^1,2^ and Zheng Ye^4^

^1^ *Department of Neurology, University of Lübeck, Lübeck 23538, Germany*

^2^ *Institute of Psychology II, University of Lübeck, Lübeck 23538, Germany*

^3^ *Institute of Neurogenetics, University of Lübeck, Lübeck 23538, Germany*

^4^ *Institute of Neuroscience, Center for Excellence in Brain Science and Intelligence Technology, Chinese Academy of Sciences, Shanghai 200031, China*

Correspondence to Z. Ye (yez@ion.ac.cn) or T.F. Münte (thomas.muente@neuro.uni-luebeck.de)

1. **No pramipexole effect on the reaction time of a delay discounting task**

We included an independent delay discounting task to examine whether pramipexole impaired digit ordering task performance selectively or task performance in general. The delay discounting task investigated the impact of delay, effort, and probability on reward-based decision-making. A formal analysis of the task is beyond the scope of this study and will be presented in a different paper.

Participants completed the delay discounting task outside the scanner immediately after the digit ordering task. They were asked to choose between a small immediate reward and a large future reward. The mean reaction times were measured under each drug and averaged across conditions (delay, effort, and probability). The mean reaction times were 2.56 s (SD=0.81) under placebo and 2.79 s (SD=1.09) under pramipexole (SFig.1). There was no significant effect of pramipexole (paired *t*-test, *t*(21)= 1.57, *p*=0.131). Pramipexole did not impair task performance in general.

SFig.1: No pramipexole effect on the reaction time of a delay discounting task. PPX, pramipexole; PLC, placebo.


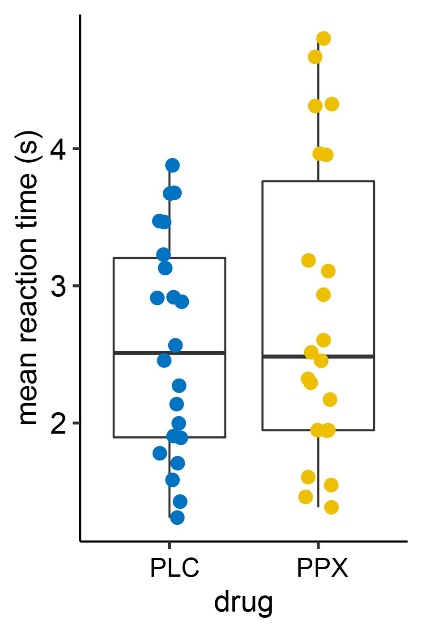


1. **Pramipexole effects on the regional activity of the right hemisphere**

We detected pramipexole effects on the regional activity of the right fronto-subthalamic and fronto-striatal pathways. The right dorsolateral prefrontal cortex (dlPFC) and right striatal regions were derived from a meta-analysis of 1091 fMRI studies on working memory (NeuroSynth) [1]. The right subthalamic nucleus (STN) region was derived from a human basal ganglia atlas [2]. Finite impulse response (FIR) timecourses of ordered and random trials were extracted from each region. SFig.2 shows pramipexole effects on the FIR timecourses of the maintenance- and manipulation-related regional activity. The right hemisphere showed tendencies similar to the left hemisphere.

SFig.2: Pramipexole effects on the regional activity of the right hemisphere. Mean FIR timecourses and SEMs of (A) the maintenance-related and (B) manipulation-related regional activity. PPX, pramipexole; PLC, placebo; dlPFC, dorsolateral prefrontal cortex; STN, subthalamic nucleus; %sc, percent signal change, asterisks, *p*<0.05.


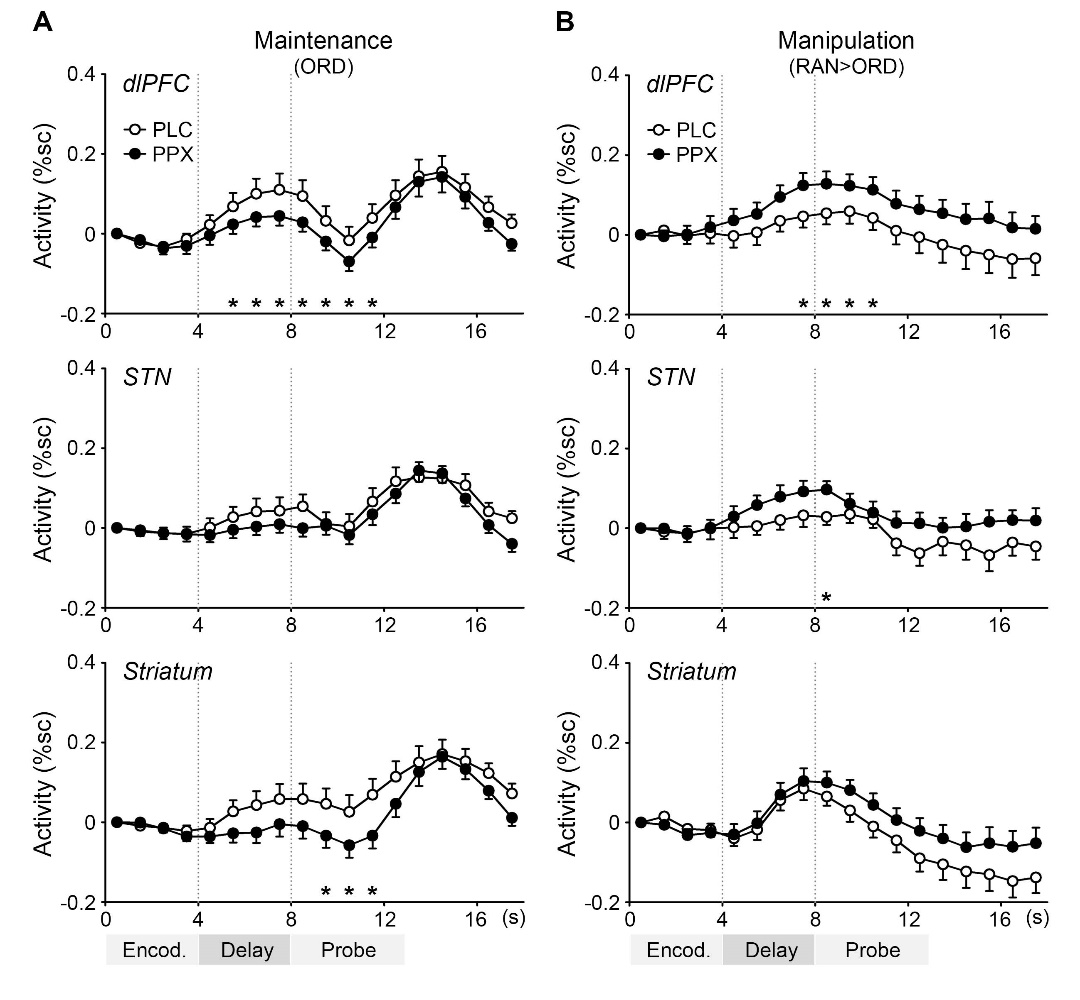


1. **References**

1 Yarkoni T, Poldrack RA, Nichols TE, Van Essen DC, Wager TD. Large-scale automated synthesis of human functional neuroimaging data. Nature Methods. 2011;8(8):665-70.

2 Prodoehl J, Yu H, Little DM, Abraham I, Vaillancourt DE. Region of interest template for the human basal ganglia: comparing EPI and standardized space approaches. Neuroimage. 2008;39(3):956-65.
